# Glasgow Public and Private 5G Performance Dataset (2025): Acquisition and Comparative Analysis

**DOI:** 10.1038/s41597-026-07600-w

**Published:** 2026-06-10

**Authors:** Ahren Hart, Hamish Sturley, Paul Mclean, Pablo Salva-Garcia, Muhammad Zeeshan Shakir

**Affiliations:** Department of Computing, Engineering and Physical Sciences, University of the West of Scotland, Paisley, PA1 2BE Scotland

## Abstract

The rollout of 5G promised gigabit speeds and sub-10 ms latency for smart cities and IoT, but real-world urban performance in the United Kingdom remains poorly documented, with few openly accessible multi-provider datasets at neighbourhood resolution. We present a publicly released dataset of 720 outdoor 5G measurements collected across 15 Glasgow neighbourhoods over three consecutive days (6–8 April 2025), using all four major UK operators (EE, Vodafone, O2, Sky Mobile) and two consumer devices (Samsung Galaxy S24 Ultra and Google Pixel 9 Pro). This dataset was collected as part of the Ayrshire 5G Innovation Region (5GIR) project to serve as a real-world urban benchmark, providing a reference for what mature public 5G performance looks like in a comparable Scottish city, against which future Ayrshire public connectivity can be assessed. City-wide averages were 670.63 Mbps download, 165.05 Mbps upload, 21.62 ms ping, and  −77.85 dBm SS-RSRP. Individual download measurements ranged from 47.84 to 1248.95 Mbps, with a coefficient of variation of 45.7%, reflecting the high variability characteristic of shared urban 5G spectrum. O2 recorded the highest median download speed (744.9 Mbps), and suburban neighbourhoods consistently outperformed the urban core. Signal strength showed negligible correlation with throughput (*r* = 0.02), highlighting the complexity of real-world 5G performance. The dataset is permanently archived and openly available under CC-BY 4.0 on Zenodo^1^ and is intended for reuse in urban propagation modelling, coverage prediction, smart-city planning, digital twinning, and as a 2025 baseline for 6G benchmarking.

## Background & Summary

The fifth generation (5G) of mobile networks offers theoretical peak download speeds exceeding 1 Gbps, latency under 10 ms, and connectivity for up to one million devices per square kilometre^2^. These capabilities support smart cities, autonomous vehicles, industrial IoT, and digital twinning through real-time data processing^3^. In the United Kingdom, public 5G services were launched in 2019 by the four major operators using non-standalone (NSA) architectures^4^. By 2023-2024, standalone (SA) deployments began, with Vodafone introducing the first nationwide SA network in June 2023, followed by Virgin Media O2 and EE in 2024^5^. Real-world performance in urban centres like Glasgow varies significantly due to shared spectrum, network congestion, site density, and topography^6^.

The evolution from 4G to 5G introduced the New Radio (NR) interface and three main service pillars: enhanced mobile broadband (eMBB), ultra-reliable low-latency communications (URLLC), and massive machine-type communications (mMTC)^2^. These are enabled by orthogonal frequency-division multiple access (OFDMA) and non-orthogonal multiple access (NOMA)^5^. Public networks prioritise broad accessibility but typically exhibit reliability between 80–95% under real-world conditions^7^.

Private (non-public) 5G networks, standardised by 3GPP as non-public networks (NPNs), use dedicated spectrum (commonly n77/n78 bands) and zero-trust security models^8^. They incorporate network slicing, multi-access edge computing (MEC), and modular architectures to achieve ultra-high reliability (up to 99.999% uptime) and consistently low latency^9^. Techno-economic studies confirm the viability of private 5G for enterprise and industrial deployments^10^. Comparative analyses show private networks outperform public networks in latency, jitter, and consistency^7^.

5G also plays a key role in sustainable IoT ecosystems. Tactile IoT capabilities enable real-time interaction required for digital twinning and smart-city automation^3^. Integration of green energy sources and energy storage with 5G networks further supports environmentally sustainable urban systems and smart grids^11^.

Despite extensive research on 5G architectures and theoretical performance, openly accessible field-measurement datasets with multi-provider coverage and neighbourhood-level resolution remain limited in the United Kingdom. Several publicly available 5G datasets exist globally: for example, the CRAWDAD/IEEE DataPort archive, including specific datasets such as the UK 4G/5G field measurement dataset by Islam et al. collected across Nottinghamshire for four major UK operators^12^, and datasets such as DoNext (a large-scale open 4G/5G measurement dataset collected via drive tests across Dortmund, Germany) provide multi-year field measurements including throughput, RSRP, and latency^13^, and national operators such as Ofcom publish aggregate coverage data for the UK^6^. However, these resources typically suffer from single-provider coverage, coarse spatial resolution, limited metric diversity, or lack of open licensing that would permit direct reuse.

A key motivation for this work is the Ayrshire 5G Innovation Region (5GIR) project, funded by the Department for Science, Innovation and Technology (DSIT) and delivered through the Digital Connectivity and Innovation Centre (DCIC) at the University of the West of Scotland. The 5GIR project is deploying and evaluating 5G infrastructure, including private network testbeds, to assess how advanced connectivity can support economic development, smart services, and digital inclusion across Ayrshire. As part of this initiative, the Glasgow public 5G dataset presented here was collected to serve as a real-world urban benchmark: a reference point illustrating what mature public 5G performance looks like across a major Scottish city, against which future Ayrshire public coverage can be compared as the region’s connectivity develops. Private 5G testbed data collected at the DCIC as part of the same project is documented in Sturley et al.^14^.

This article addresses the open-data gap through empirical analysis of public 5G performance in Glasgow based on 720 measurements across 15 neighbourhoods, revealing city-wide averages of 670.63 Mbps download, 165.05 Mbps upload, 21.62 ms ping, and  − 77.85 dBm signal strength. Download speeds ranged from 47.84 to 1248.95 Mbps across the full dataset. The data show consistent spatial variations, with suburbs outperforming urban cores due to lower congestion.

This work contributes:

- A multi-provider, neighbourhood-resolution public 5G performance dataset for a major UK city, openly released under CC-BY 4.0 for immediate reuse,
- empirical evidence of spatial performance variations and the consistent suburban advantage in real-world conditions,
- a 2025 urban baseline for the Ayrshire 5GIR project, providing a reference for what mature public 5G performance looks like in a comparable Scottish city as Ayrshire’s own connectivity develops,
- full methodological transparency, including devices, tools, and stratified sampling protocol, to ensure reproducibility.

The public 5G dataset, with complete documentation, is intended for reuse in urban propagation modelling, coverage prediction, smart-city planning, digital twinning, and machine-learning applications. It serves as a 2025 baseline for future 6G measurement campaigns and as a reference benchmark for the Ayrshire 5GIR project. No portion of this dataset has been published previously.

## Methods

This section outlines the data collection and analysis procedures employed in this study to ensure reproducibility and methodological rigour. The approach focuses on empirical measurements of both public and private 5G networks, drawing on standardised performance evaluation techniques to capture real-world metrics such as throughput, latency, signal strength, and reliability. All tests were conducted in controlled yet representative environments, prioritising authenticity over simulated ideals to align with practical applications in IoT, smart cities, and digital twinning.

### Public 5G Data Collection

To benchmark real-world public 5G performance, a dataset was compiled from 720 real-world measurements across 15 neighbourhoods in Glasgow, Scotland: Bearsden, Cathcart, Dennistoun, Drumchapel, Easterhouse, Glasgow City Centre, Govan, Govanhill, Hillhead, Maryhill, Merchant City, Partick, Pollok, Shawlands, and Springburn. These locations were selected to represent a mix of urban densities, from high-congestion central areas to suburban zones, enabling analysis of spatial variations influenced by infrastructure and topography. Locations were stratified by density using Ordnance Survey OpenData to ensure balanced representation.

Measurements were collected over a three-day period from 6–8 April 2025, using the Ookla Speedtest application for download/upload speeds and ping latency, in conjunction with the Cellular-Z app to verify active 5G NR connections and capture SS-RSRP signal quality metrics. The Ookla Speedtest application was configured to automatically select the nearest available server based on ping latency (default server-selection mode), ensuring consistent and reproducible conditions. All public 5G measurements were conducted outdoors with the device held stationary (not in motion) at each designated neighbourhood location. Tests were carried out between 08:30 and 19:53 each day, spanning both morning and evening periods to capture variation in network load throughout the day. The Cellular-Z SS-RSRP reading displayed immediately prior to each Speedtest was manually recorded and matched to the corresponding Speedtest result using the UTC timestamp logged by both applications; readings were only paired when both timestamps were within a 30-second window, ensuring accurate co-location of radio and throughput metrics. This dual-tool approach allowed for cross-validation of performance data under real-user conditions, as informed by the key differences in 4G and 5G characteristics outlined in Table 1.

**Table 1:** High-Level Comparison of 4G and 5G Characteristics.

Characteristic	4G (LTE-Advanced)	5G (New Radio)
Peak Data Rate	1 Gbps	10-20 Gbps
Latency	20–50 ms	~1 ms
Device Density	~100,000/km^2^	1 million/km^2^
Core Architecture	Monolithic (EPC)	Service-Based (5GC)
Key Service Pillars	Mobile Broadband	eMBB, URLLC, mMTC
Signal Quality Metrics	RSRP, RSRQ, SINR	SS-RSRP, SS-RSRQ, SS-SINR
Current Coverage	~95% (Scotland)	~29–51% (Scotland)

Tests were performed on two 5G-enabled consumer devices: the Samsung Galaxy S24 Ultra and Google Pixel 9 Pro (both Android-based, compatible with Cellular-Z). Tests were conducted across four major network providers: EE, Vodafone, O2, and Sky Mobile, to reflect the dominant market landscape in the UK. Data gathering followed a structured protocol: 2 data points were collected per provider per device per location per day, yielding a total of 720 measurements (2 × 4 × 2 × 15 × 3 = 720). Each of these 720 rows in the dataset represents the mean of 5–10 repeated Speedtest runs performed consecutively at the same location, provider, device, and time slot (a single test session); this within-session averaging reduces the impact of momentary network fluctuations on each recorded data point. The 720 sessions spanned different times of day (morning, midday, and afternoon/evening slots across the three collection days) to partially capture diurnal variation. Each session consisted of a full test cycle (download speed, upload speed, latency, and SS-RSRP capture) under stationary outdoor conditions.

The data collection protocol is summarised in Table 2.

**Table 2:** Data Collection Protocol.

Parameter	Description
Devices	Samsung Galaxy S24 Ultra, Google Pixel 9 Pro
Tools	Ookla Speedtest (for speeds and latency), Cellular-Z (for network verification and signal strength)
Networks	EE, Vodafone, O2, Sky Mobile
Metrics Collected	Download Speed (Mbps), Upload Speed (Mbps), Ping (ms), SS-RSRP (dBm)
Dates	6–8 April 2025
Locations	15 neighbourhoods of Glasgow (listed above)
Measurements per Combination	2 data points per provider per device per location per day
Total Measurements	720 (under varying conditions)

Raw data were exported from both apps as CSV files and cross-verified for consistency (e.g., ensuring 5G NR mode was active via Cellular-Z logs). All records were validated against domain-based physical bounds prior to analysis: download and upload speeds must be >0 Mbps, ping must be >0 and <500 ms, and SS-RSRP must fall within the 3GPP TS 38.133 valid range ( −44 to −140 dBm); all 720 records passed without exception and no records were removed. Data were then analysed for averages, distributions, and correlations using Python with pandas and NumPy libraries. The Cellular-Z application also surfaces additional Android radio metrics (SS-RSRQ, SS-SINR, ARFCN, and band information); however, these values were not consistently available across all provider/device combinations during the measurement period and are therefore not included in the released dataset. Future measurement campaigns are encouraged to capture this wider set of metrics to enrich longitudinal analyses.

### Private 5G Data Collection

The private 5G testbeds described in this section are part of the Ayrshire 5G Innovation Region (5GIR) project, delivered through the Digital Connectivity and Innovation Centre (DCIC) at the University of the West of Scotland and funded by the Department for Science, Innovation and Technology (DSIT). The DCIC hosts two operational private 5G testbeds which have been used to develop, demonstrate, and evaluate advanced connectivity use cases relevant to Ayrshire’s digital connectivity ambitions. These testbeds are referenced here to provide context for the motivation behind the Glasgow public 5G dataset which was collected as a real-world urban benchmark to illustrate what mature public 5G performance looks like in a comparable Scottish city, informing expectations for what Ayrshire could achieve as public 5G coverage develops in the region. The design, deployment, and performance evaluation of these private 5G testbeds is documented in Sturley *et al*.^14^. The MPN components are detailed in Table 3, and the O-RAN components are in Table 4.

**Table 3:** Components of Vodafone and Ericsson MPN.

Component	Description
Ericsson EP5G v1.1 Core	Supports up to 100 users with 1Gbps throughput.
Ericsson BBU 6631 Baseband Unit	Provides six radio interfaces with eCPRI support.
Indoor Radio Units (IRU 8848) and NR Dot 4459 (B77D, 4T4R)	Operates in the n77 band with 100 MHz bandwidth.
Dell R640 Network Controllers, Ericsson GNSS Sync Module and Antenna	
Self-service portal and network management tools	for real-time monitoring and control.

**Table 4:** Components of BubbleRan O-RAN.

Component	Description
MX-PDK Compute Nodes (x3)	High-performance workstations with 25Gbps network interfaces.
Lite-On FlexiFI O-RU n78 (x2)	4 × 4 MIMO, 100MHz indoor radio units
FibroLAN Falcon-RX Switch + GNSS	Provides synchronisation through time-aware networking.
Quectel RM500 Modules (x5)	
Software Stack	OpenAirInterface, FlexRIC, ODIN, Kubernetes, Docker, Grafana, and Timescale DB.

#### Vodafone and Ericsson Mobile Private Network (MPN)

#### BubbleRAN Open Radio Access Network (O-RAN)

## Data Records

The dataset is permanently archived at https://doi.org/10.5281/zenodo.20465872^1^ under CC-BY 4.0.

The repository contains a single primary data file, Glasgow5GDataSet.xlsx, which is organised into four worksheets:

- glasgow_5g_2025-04-06: 240 raw measurement rows for 6 April 2025 (08:30–19:49)
- glasgow_5g_2025-04-07: 240 raw measurement rows for 7 April 2025 (08:30–19:49)
- glasgow_5g_2025-04-08: 240 raw measurement rows for 8 April 2025 (08:30–19:53)
- Averaged Dataset: per-location means aggregated across all three days

Each raw worksheet contains 8 columns, described in Table 5. The Averaged Dataset sheet contains per-neighbourhood means for download speed, upload speed, ping, and signal strength, as reproduced in Table 6.

**Table 5:** Columns in the raw daily worksheets (Glasgow5GDataSet.xlsx).

Column name	Description	Unit	Example value
Timestamp	UTC timestamp of measurement	ISO 8601	2025-04-06T08:30:00Z
Location	Glasgow neighbourhood	string	Springburn
Network Provider	Mobile network operator	string	Vodafone
Test Device	Device used for measurement	string	Samsung Galaxy S24 Ultra
Signal Strength (dBm)	SS-RSRP; range in dataset: −97 to −56 dBm	dBm	−72
Download Speed (Mbps)	Download throughput; range: 47.8–1249.0 Mbps	Mbps	907.32
Upload Speed (Mbps)	Upload throughput; range: 11.8-310.3 Mbps	Mbps	176.78
Ping (ms)	Round-trip latency; range: 3.9–43.2 ms	ms	17.26

**Table 6:** Averaged Real-World 5G Network Performance Across Glasgow Neighbourhoods.

Location	Download Speed (Mbps) Mean	Upload Speed (Mbps) Mean	Ping (ms) Mean	Signal Strength (dBm) Mean
Bearsden	633.88	161.43	20.62	−77.62
Cathcart	681.34	164.10	20.79	−77.81
Dennistoun	648.51	156.63	23.87	−77.06
Drumchapel	682.05	154.82	22.47	−78.31
Easterhouse	707.79	173.77	21.88	−77.94
Glasgow City Centre	638.76	174.35	20.99	−76.31
Govan	704.00	166.20	21.95	−79.38
Govanhill	680.19	161.16	22.28	−78.44
Hillhead	600.23	157.31	22.44	−78.00
Maryhill	631.37	171.87	20.53	−76.75
Merchant City	688.23	156.06	21.19	−78.62
Partick	677.89	165.23	19.73	−77.88
Pollok	699.40	176.15	22.42	−77.62
Shawlands	684.28	180.90	21.16	−77.88
Springburn	701.56	155.71	21.99	−78.19
Glasgow Average	670.63	165.05	21.62	−77.85

The repository also contains:

- README.md: complete codebook, neighbourhood descriptions, licence, and citation guidance
- License.md: licence text

Column definitions are provided in Table 5.

This dataset is designed for immediate reuse in urban network modelling, coverage prediction, smart-city planning, digital twinning, machine-learning baselines, and as a 2025 reference point for future 6G measurement campaigns.

## Data Overview

Across the 720 measurements, city-wide averages were 670.63 Mbps download, 165.05 Mbps upload, 21.62 ms ping, and  − 77.85 dBm SS-RSRP. Download speeds ranged from 47.8 to 1249.0 Mbps; upload from 11.8 to 310.3 Mbps; ping from 3.9 to 43.2 ms; and signal strength from  − 97 to  − 56 dBm. Per-neighbourhood means across all four metrics are summarised in Table 6.

**Fig. 1:**
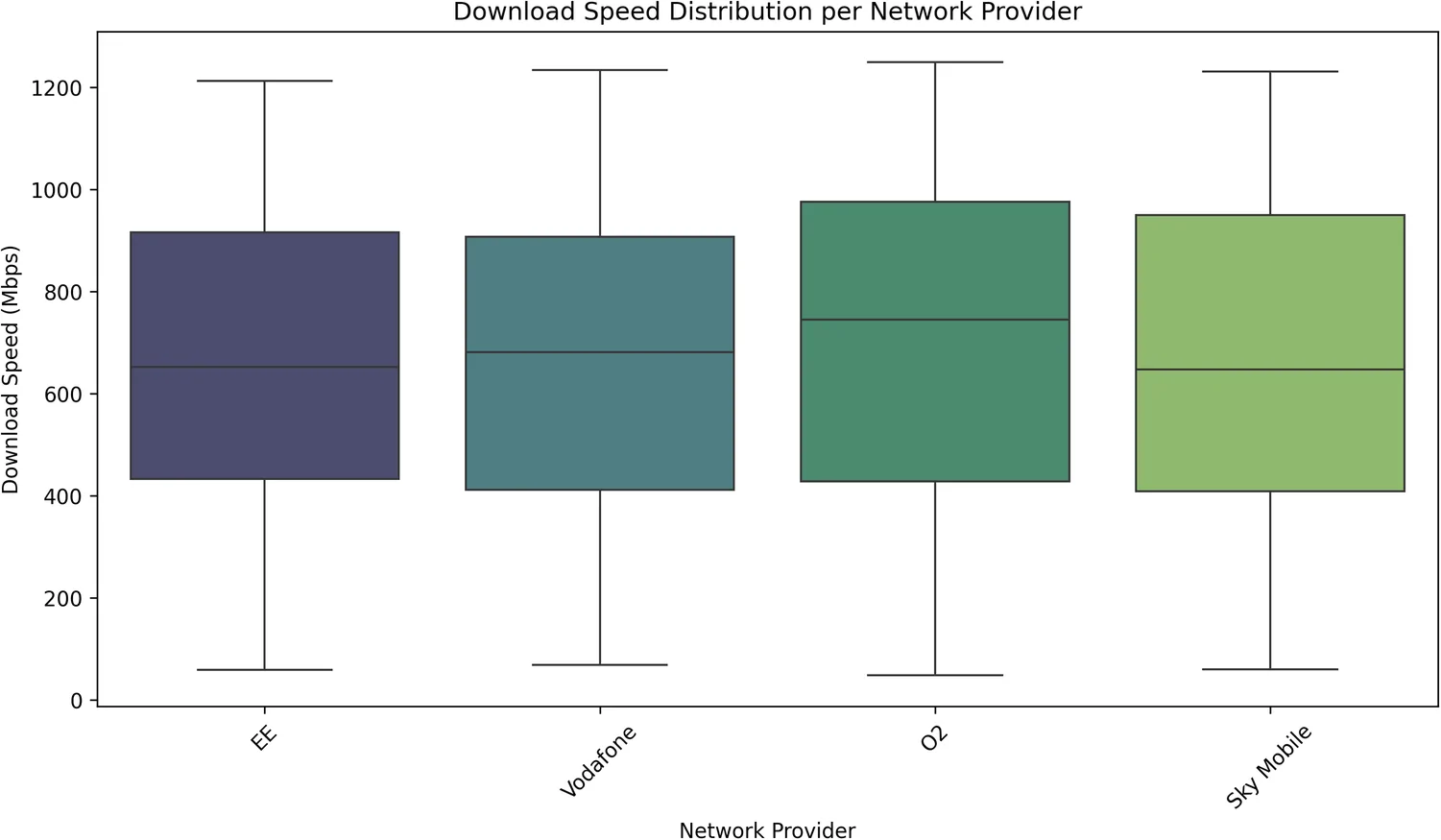
Download speed distribution per network provider (N = 720). Box plots show median, interquartile range, and whiskers to the data extremes.

Download speed varied considerably across providers. Figure 1 shows the full distribution of download speeds per provider as a box plot. O2 recorded the highest median download speed (744.9 Mbps), followed by Vodafone (681.3 Mbps), EE (652.6 Mbps), and Sky Mobile (647.2 Mbps). All providers exhibited wide interquartile ranges, reflecting the high measurement-to- measurement variability inherent in shared public 5G spectrum.

**Fig. 2:**
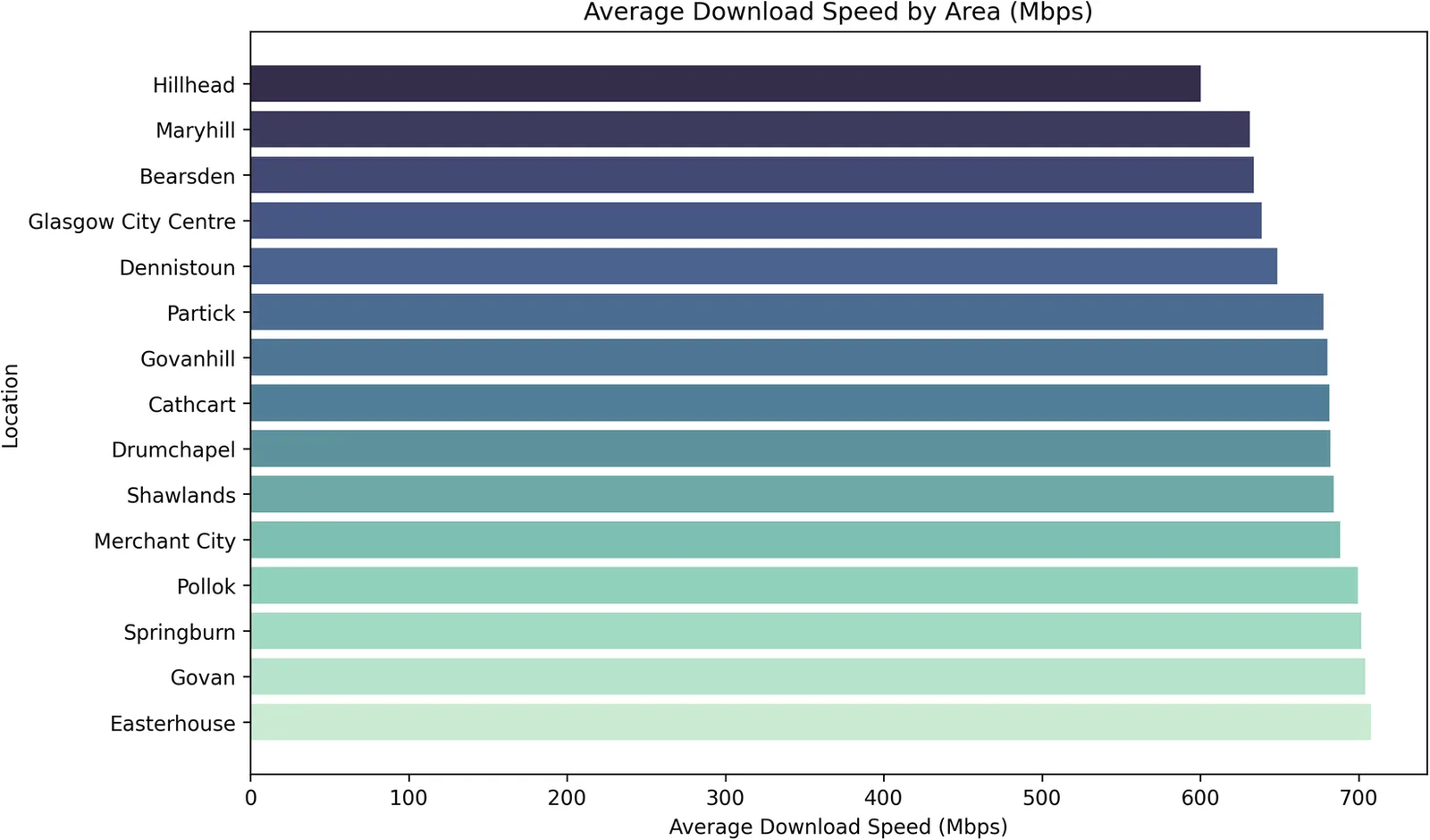
Average download speed by neighbourhood (N = 48 measurements per location), ranked ascending. Colour gradient reflects relative performance.

Spatial variation in download performance is visible in Fig. 2, which ranks all 15 neighbourhoods by mean download speed. The three highest-performing locations were Easterhouse (707.79 Mbps), Govan (704.00 Mbps), and Springburn (701.56 Mbps), while the lowest were Hillhead (600.23 Mbps), Maryhill (631.37 Mbps), and Bearsden (633.88 Mbps). This suburban-over-urban pattern is consistent with lower network congestion in outer areas.

**Fig. 3:**
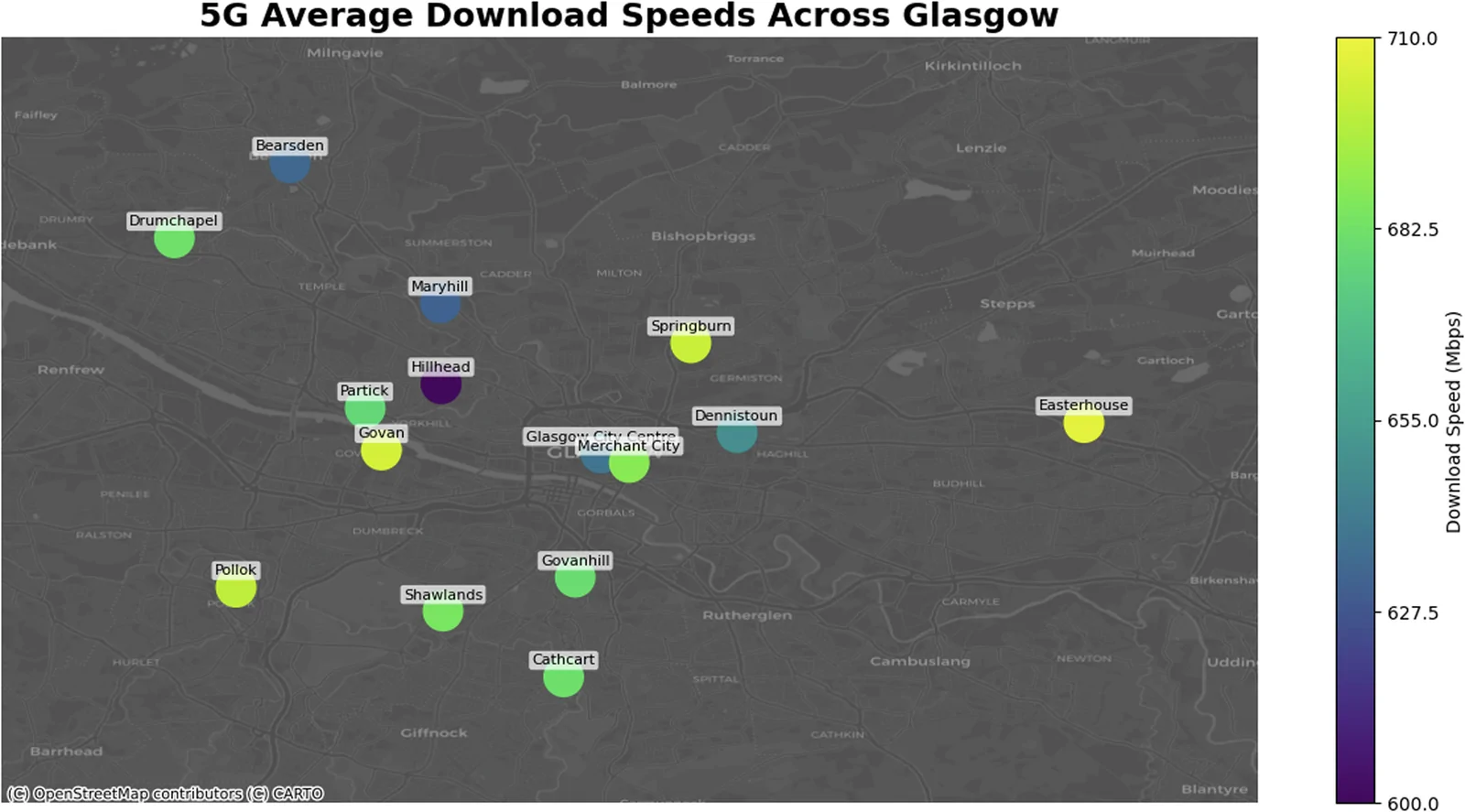
Geospatial visualisation of average 5G download speed across 15 Glasgow neighbourhoods. Colour scale ranges from 600 Mbps (purple) to 710 Mbps (yellow).

The geographic distribution of download performance is further illustrated in Fig. 3, a geospatial heat map overlaid on Glasgow. Outer neighbourhoods (Easterhouse, Pollok, Springburn) are rendered in yellow/green indicating higher speeds, while the denser urban core (Hillhead, Glasgow City Centre) appears in blue/purple indicating lower speeds.

**Fig. 4:**
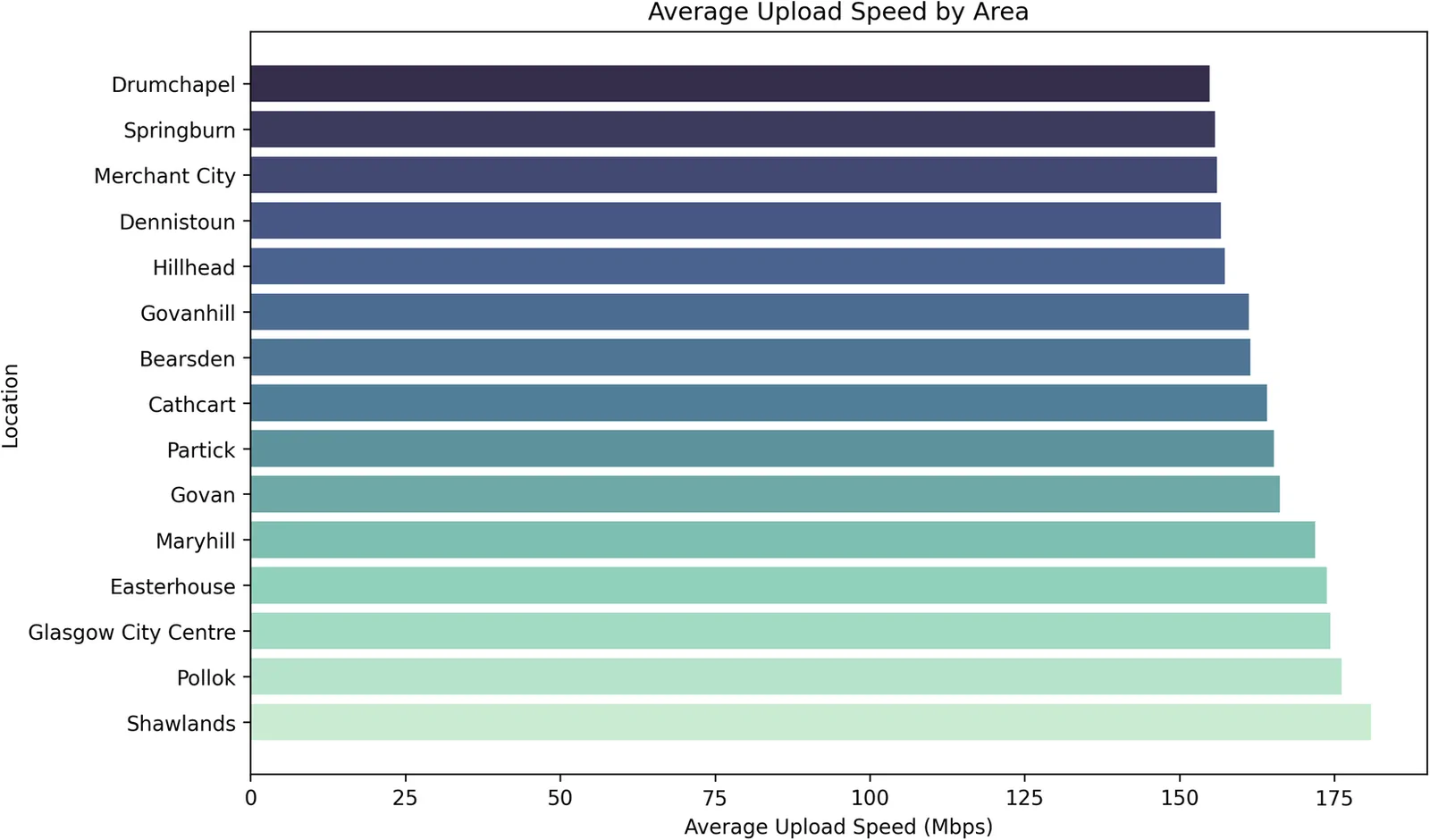
Average upload speed by neighbourhood, ranked ascending.

Upload performance followed a broadly similar geographic pattern to download, as shown in Fig. 4. Shawlands recorded the highest mean upload speed (180.90 Mbps) and Drumchapel the lowest (154.82 Mbps). Upload variability across locations was considerably smaller than for download, with all neighbourhood means falling between 154 and 181 Mbps.

**Fig. 5:**
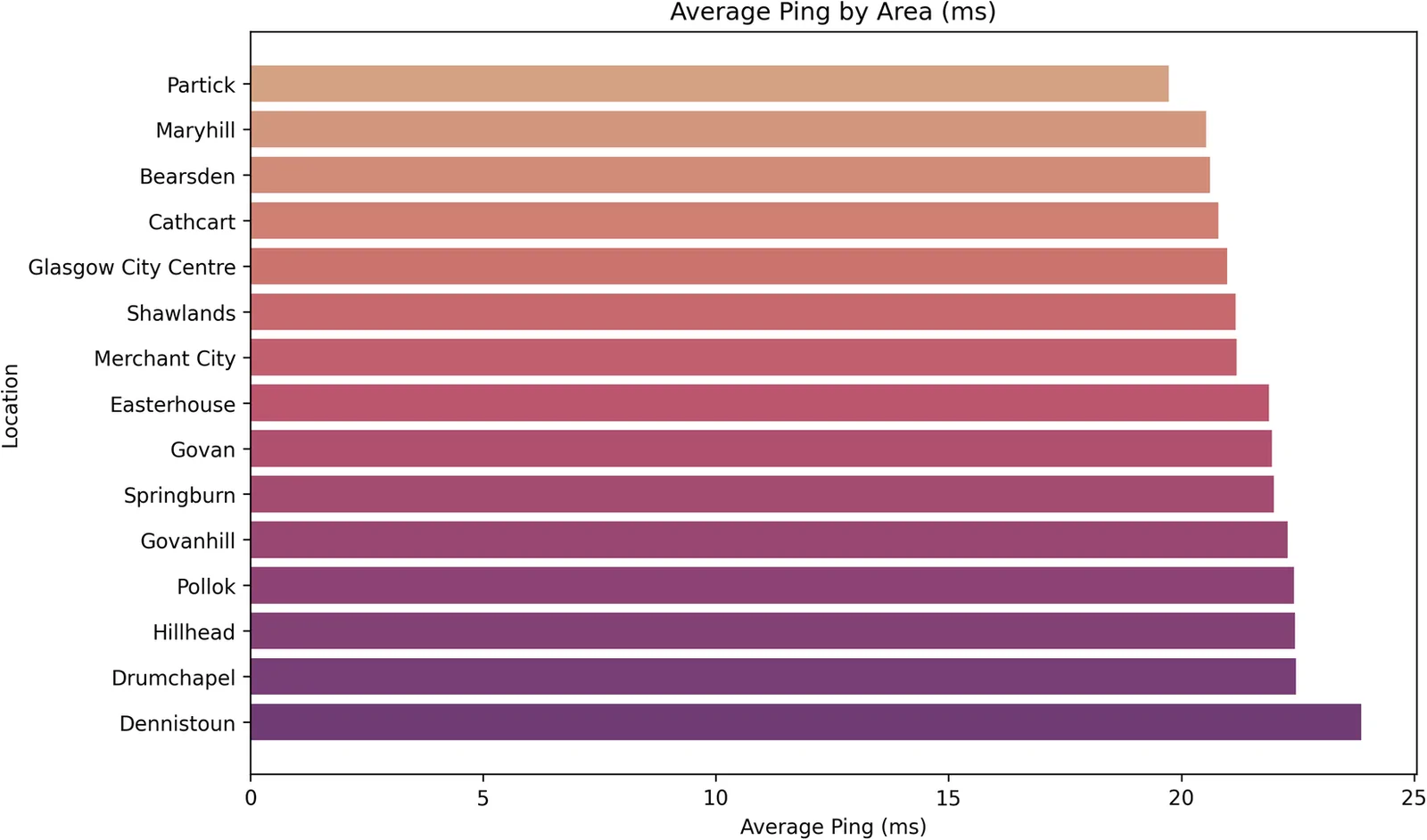
Average ping latency by neighbourhood, ranked ascending (lower values indicate better latency performance).

Ping latency by neighbourhood is shown in Fig. 5. Partick recorded the lowest mean latency (19.73 ms), followed by Maryhill (20.53 ms) and Bearsden (20.62 ms). Dennistoun had the highest mean latency (23.87 ms). Notably, Glasgow City Centre did not rank among the lowest-latency areas despite its dense infrastructure, suggesting congestion effects on latency during the measurement window.

**Fig. 6:**
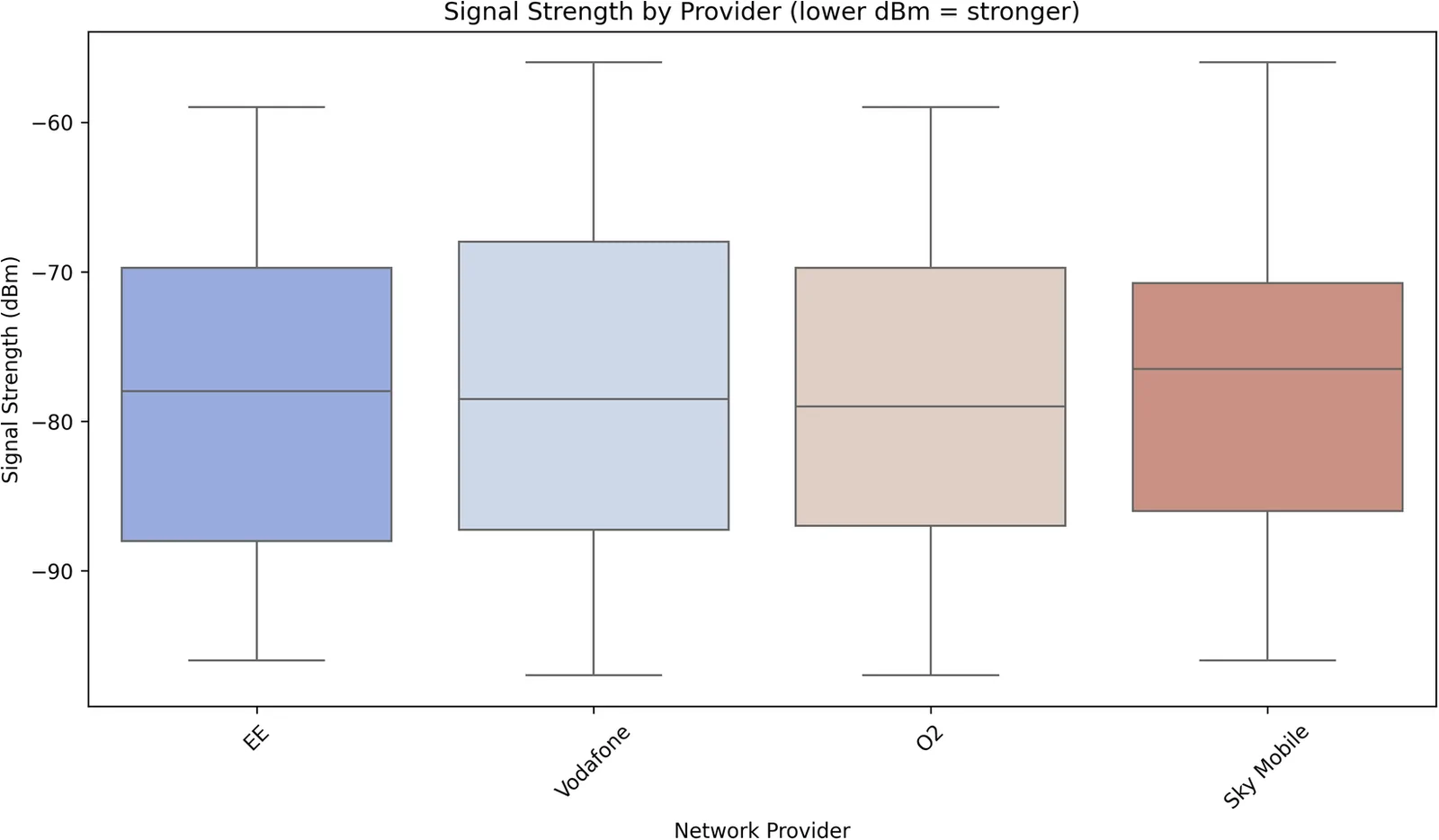
SS-RSRP signal strength distribution by network provider. Note: lower dBm values (more negative) indicate weaker signals; less negative values indicate stronger signals.

Signal strength (SS-RSRP) distributions by provider are presented in Fig. 6. Sky Mobile recorded the strongest median SS-RSRP (−76.5 dBm), while O2 recorded the weakest (−79.0 dBm). All four providers exhibited broadly similar distributions, with medians clustered between −76.5 and −79.0 dBm, confirming comparable mid-band signal penetration across providers in the measurement locations.

**Fig. 7:**
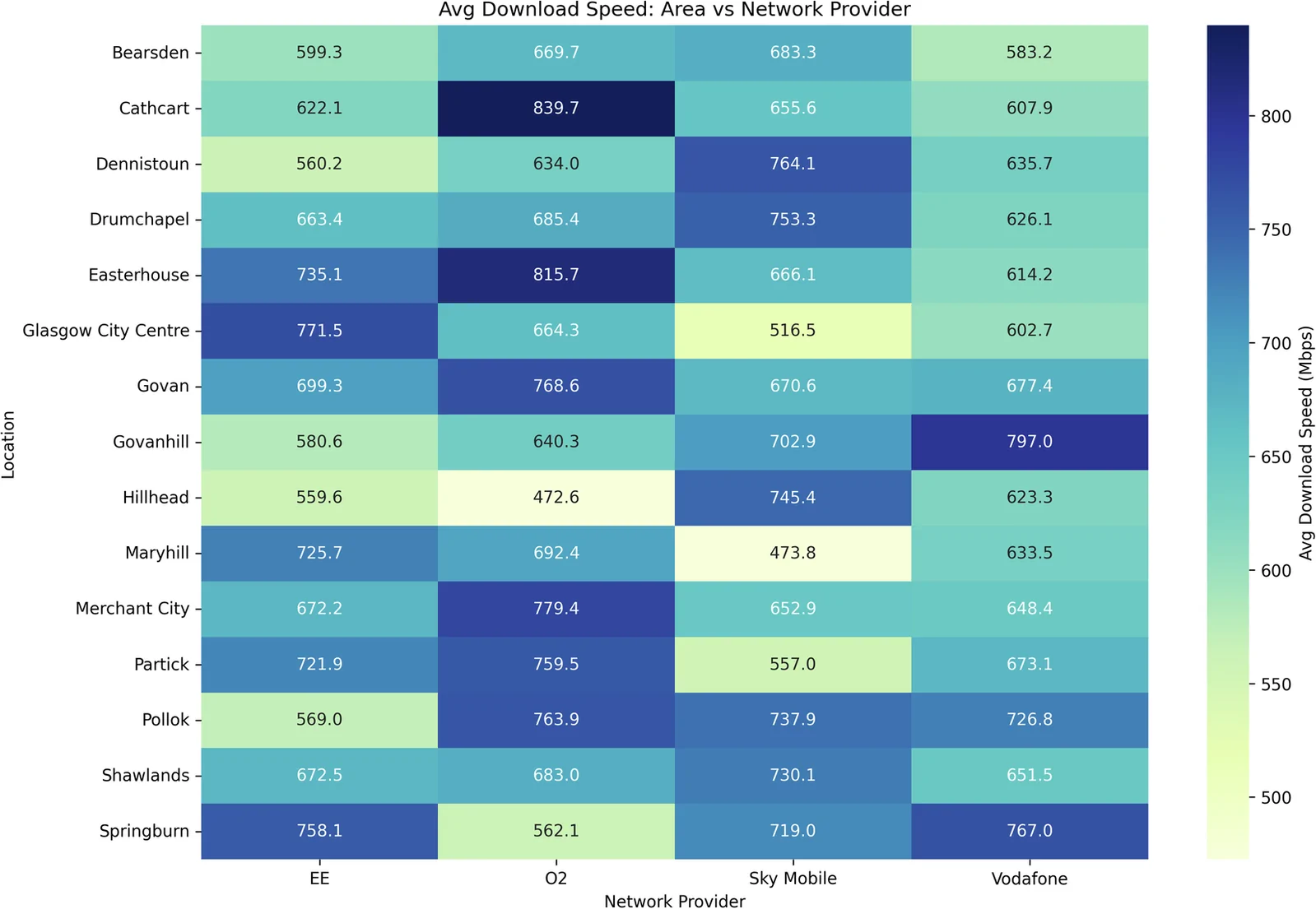
Heat map of average download speed (Mbps) across all 15 neighbourhood and 4 provider combinations. Each cell represents the mean of 12 measurements (2 devices  × 2 data points  × 3 days).

Finally, Fig. 7 presents a combined area-by-provider heat map of mean download speeds, allowing direct comparison across all 60 location-provider combinations. Notable cells include Cathcart/O2 (839.7 Mbps) and Govanhill/Vodafone (797.0 Mbps) as the highest-performing combinations, and Maryhill/Sky Mobile (473.8 Mbps) and Hillhead/O2 (472.6 Mbps) as the lowest.

## Technical Validation

### Data Integrity and Validity Checks

Each record in the dataset was validated against published physical and standards-based bounds prior to analysis. Specifically: download and upload speeds must be >0 Mbps (a zero or negative value is physically impossible for a completed test); ping latency must be >0 ms and <500 ms (values exceeding 500 ms indicate a failed or dropped connection rather than a valid 5G measurement); and SS-RSRP must fall within the valid range defined by 3GPP TS 38.133 (−44 to −140 dBm). All 720 records passed every bound without exception: download speeds ranged from 47.84 to 1248.95 Mbps, upload from 11.79 to 310.27 Mbps, ping from 3.93 to 43.21 ms, and SS-RSRP from −97 to −56 dBm. No records were removed. The low end of the download range (e.g. sub-100 Mbps readings at Bearsden) represents genuine real-world variability. Bearsden exhibited a standard deviation of 359 Mbps across its 48 measurements, reflecting authentic fluctuations in congestion and radio conditions rather than measurement error. Active 5G NR connections were additionally confirmed for every record via the Cellular-Z network-type indicator prior to each Speedtest run.

### Timestamp Cross-Validation

Speedtest and Cellular-Z readings were matched using UTC timestamps. Only pairs with timestamps within a 30-second window were retained, ensuring that each SS-RSRP value in the dataset corresponds to the radio conditions present during its associated throughput test. This pairing approach is reflected in the column structure of the raw worksheets described in Table 5.

### Statistical Checks

The coefficient of variation (CV) for download speed, calculated as the standard deviation (306.68 Mbps) divided by the mean (670.63 Mbps) multiplied by 100, is 45.7%. This high CV is expected for a shared urban 5G network where individual measurements span from 47.8 to 1249.0 Mbps, reflecting real congestion and propagation variability across 15 diverse locations, 4 providers, 2 devices, and 3 days. The Pearson correlation between SS-RSRP and download speed across all 720 measurements is *r* = 0.02, confirming that raw signal strength is not a meaningful predictor of throughput on mid-band 5G networks, a finding consistent with comparable studies^7^. Provider-level distributions (Figure 1) and the area-by-provider heat map (Figure 7) were both inspected for anomalous skewness or outlier cells; all values are within plausible ranges. Sky Mobile’s signal strength performance (Figure 6) closely mirrors O2’s, as expected given that Sky Mobile is an MVNO operating on O2’s physical radio infrastructure; the small observed difference of approximately 2.5 dBm in median SS-RSRP is within normal device-level reporting variability.

### Measurement Limitations

The three-day collection window (6–8 April 2025) does not capture seasonal or longitudinal network load trends, as acknowledged in Table 2. Only two consumer device models (Samsung Galaxy S24 Ultra, Google Pixel 9 Pro) were used, which may introduce hardware-specific biases in both Speedtest results and SS-RSRP reporting. All public measurements were conducted outdoors in stationary conditions; deep-indoor and rural environments were not assessed. The high coefficient of variation for download speed (45.7%) reflects genuine real-world variability across locations, providers, and time slots rather than data quality issues; all values were confirmed within 3GPP physical bounds as described above. Only SS-RSRP was consistently extractable across all provider/device combinations; additional radio metrics (SS-RSRQ, SS-SINR) were not reliably available and are therefore excluded from the released dataset. Future campaigns are encouraged to capture this wider set of metrics to enrich longitudinal analyses.

### Ethical Approval and Consent

This study did not involve human or animal subjects, or personal data. No ethical approval or informed consent was required.

## Data Availability

The public 5G dataset is permanently archived on Zenodo and freely available under a Creative Commons Attribution 4.0 International (CC-BY 4.0) licence at: https://doi.org/10.5281/zenodo.20465872.The repository contains: - README.md — dataset description, column codebook, licence, and citation instructions Private 5G laboratory measurements are not released.

## Code availability

Basic analysis of the dataset can be performed directly in Excel or with any spreadsheet software. A single reproducible Python script (FigureGeneration.py) is used to generate all seven figures in this paper. The script uses pandas, matplotlib, seaborn, geopandas, shapely, contextily, pillow, and numpy. It is available at the Zenodo repository alongside the dataset: https://doi.org/10.5281/zenodo.20465872.

A minimal dataset loading example is provided in the README.md.
